# Is fracture management merely a physical process? Exploring the psychological effects of internal and external fixation

**DOI:** 10.1186/s13018-024-04655-6

**Published:** 2024-04-08

**Authors:** Qiyu Jia, Zhenlei Peng, Anqi Huang, Shijie Jiang, Wen Zhao, Zengru Xie, Chuang Ma

**Affiliations:** 1 Department of Trauma Orthopedics, The First Affiliated Hospital of Xinjiang Medical University, Urumqi, Xinjiang China; 2 Xinjiang Clinical Research Center for Mental Health, The First Affiliated Hospital of Xinjiang Medical University, Urumqi, Xinjiang China; 3 Nanjing Brain Hospital, Clinical Teaching Hospital of Medical School, Child Mental Health Research Center, Nanjing University, Nanjing, China; 4 Department of Orthopedics, Beijing Aerospace General Hospital, Beijing, China; 5 Department of Orthopedics, The First Affiliated Hospital of Xinjiang Medical University, Urumqi, Xinjiang China

**Keywords:** Distal radius fractures, Internal fixation, External fixation, Depression, Anxiety, Quality of life

## Abstract

**Background:**

Internal and external fixation are common surgical procedures for treating fractures. However, the impact of different surgical approaches (including internal and external fixations) on patients’ psychological status and Quality of Life (QoL) is rarely examined. Herein, we aimed to investigate the effects of internal and external fixation on anxiety, depression, insomnia, and overall mental and physical health in Distal Radius Fractures (DRF) patients.

**Methods:**

We performed a retrospective study on 96 fracture patients who underwent internal fixation (57 patients) or external fixation (39 patients). The Visual Analog Scale (VAS), the Hospital Anxiety and Depression Scale (HADS), the Athens Insomnia Scale (AIS), and the Medical Outcomes Study Short Form 36 (SF-36) questionnaire were used to assess the patients’ pain, anxiety, depression, sleep, and QoL before surgery and at seven days, one month, and three months post-surgery.

**Results:**

The VAS scores were significantly lower in the Internal Fixation Group (IFG) than in the External Fixation Group (EFG) on the seventh day and one month postoperatively (*P* < 0.05). Although both groups showed no significant anxiety, depression, or insomnia before surgery (*P* > 0.05), the EFG showed significantly higher HADS-A, HADS-D, and AIS scores than the IFG at seven days and one and three months postoperatively (*P* < 0.05). Additionally, changes in HADS-A, HADS-D, and AIS scores were most significant at day seven post-surgery in the EFG (*P* < 0.05). Furthermore, no significant difference was found between the two groups in the average Physical Component Summary (PCS) and Mental Component Summary (MCS) scores before surgery (*P* > 0.05). However, both groups showed positive changes in PCS and MCS scores at postoperative day seven and one and three months postoperatively, with the IFG having significantly higher average PCS and MCS scores compared to the EFG (*P* < 0.05).

**Conclusion:**

Compared to external fixation, internal fixation did not significantly impact patients’ emotions regarding anxiety and depression in the early postoperative period, and physical and mental health recovery was better during the postoperative rehabilitation period. Furthermore, when there are no absolute indications, the impact on patients’ psychological well-being should be considered as one of the key factors in the treatment plan during surgical approach selection.

## Introduction

In our clinic, we encountered a patient who said, “I don’t want to continue with the treatment anymore, please remove the frame, i’m becoming depressed.” This patient had been wearing an external fixation device for six months. This incident astonished us and made us realize we could have overlooked some critical aspects in our previous treatment approaches.

As common injuries, fracture-induced physical and psychological distress may impact patients’ Quality of Life (QoL) and daily functioning [1–3]. Treatment methods for fractures have notably progressed with continuous advancements in medical technology. Internal and external fixations are the two commonly used surgical approaches for stabilizing the fracture site and promoting healing and recovery [4–8]. However, besides the physical recovery process, the psychological well-being of fracture patients is the other important dimension that should be carefully considered. Recognizing the impact of psychological factors on health outcomes, the modern medical model has evolved from the traditional biomedical to the biopsychosocial model. The iatrogenic secondary psychological harm caused by medical interventions is one phenomenon that many practitioners have overlooked but constantly occurs. According to research [9–11], medical interventions such as surgery may have unintended effects on individuals’ psychological well-being and QoL besides promoting healing and alleviating pain. Specifically, some externally visible implants, such as external fixation devices, could potentially impact patients’ mental health [12, 13]. Furthermore, multiple studies [13–16] recently reported that patients who undergo external fixation surgery may experience adverse emotional reactions, including tension, worry, irritability, depression, and low self-esteem. In addition to affecting the patients’ recovery process, these adverse emotional reactions can prolong the rehabilitation period. Furthermore, a study [14] previously reported that psychological health may deteriorate to a moderate to severe extent in up to 50% of adolescents on Ilizarov fixators, potentially leading to suicidal tendencies. Presently, there are no systematic studies on the effects of internal fixation and external fixation surgeries on patients’ postoperative psychological health. Consequently, there is an urgent need to explore the impact of fixation methods on the postoperative psychological status of fracture patients.

This study aims to evaluate the impact of two surgical approaches on patients’ psychological health (regarding anxiety, depression, and insomnia) and QoL post-surgery. Specifically, we aim to establish a new perspective on the comprehensive treatment of fracture patients, offering them more hope and opportunities during recovery. Moreover, our study findings may guide healthcare professionals, allowing them to better recognize and address psychological challenges that fracture patients may face during treatment.

## Materials and methods

### Study design and participants

The population for this retrospective study consisted of 96 patients who underwent either external or internal fixation surgery at the First Affiliated Hospital of Xinjiang Medical University, between June 2021 and May 2023. Among them, 57 and 39 patients underwent internal fixation and external fixation surgeries, respectively. The 39 patients who underwent external fixation surgery used the ORTHOFIX wrist external fixation frame system. The ORTHOFIX system features a ball-and-socket joint slider mechanism that allows early functional exercises for patients while wearing the external fixator. The inclusion criteria were as follows: (1) Patients aged 18–60 years; (2) Patients had closed, fresh, and unstable distal radius fractures (DRF) classified as AO/OTA types B2, B3, C1, and C2; (3) Patients who have already undergone internal or external fixation surgery in the hospital; (4) Patients in which none of the internal or external fixation devices had been removed at three months post-surgery; (5) Patients who can read traditional Chinese; and (6) Patients without physical limitations that could prevent them from completing the self-administered questionnaire. The exclusion criteria were as follows: (1) History of previous surgery on the affected limb; (2) Multiple and intra-articular fractures; (3) Patients with infection at the fracture site; (4) Presence of combined autoimmune diseases, malignancies, and so on; (5) Patients with pathological fractures; (6) Patients with other significant medical illnesses and psychiatric history; (7) Patients with incomplete clinical data; and (8) Withdrawal from the study or inability to complete the scale assessments.

### Data collection

The Patient Information Form, the Visual Analog Scale (VAS) pain scores [17], the Hospital Anxiety and Depression Scale (HADS) [18, 19], the Athens Insomnia Scale (AIS) [20, 21], and the Medical Outcomes Study Short Form 36 (SF-36) [22–24] were used to collect patient mood data through face-to-face interviews. Review of patient records indicated that information on the study’s purpose and goals had been documented, and written informed consent was obtained accordingly. Patient information was collected at admission, on the day of surgery, and at discharge. The data were then compiled into a comprehensive patient information form. Furthermore, patients underwent other assessments upon admission. Specifically, the VAS, HADS, AIS, and SF-36 scales were completed at admission, on postoperative day seven, and at one and three months postoperatively.

### Data collection tools

#### Patient information form

The questionnaire comprises 14 questions aimed at evaluating various patient characteristics, including age, gender, body mass index (BMI), marital status, education level, family location, cause of injury, side of hand, AO classification [25], interval from injury to surgery, operative time, incision length, operative blood loss, and length of hospital stay.

#### VAS

The VAS, which rates pain on a scale of 1 (no pain) to 10 (worst pain), with higher scores indicating more severe pain, was used to assess pain levels [17].

#### HADS

We used HADS to evaluate perioperative anxiety and depression. It is a patient-centered survey comprising seven items each for anxiety and depression, and respondents rate each item on a scale of 0 to 3. For each subscale total score, 0–7 indicates no symptoms, 8–10 suggests mild symptoms, and 11–21 shows the presence of anxiety or depression. Patients with a total score ≥ 11 were considered anxious or depressed [19]. The Chinese version of HADS has demonstrated good internal consistency, with Cronbach’s alphas of 0.85, 0.79, and 0.79 for the full scale, depression subscale, and anxiety subscale, respectively [18].

#### AIS

The AIS is a reliable tool for assessing subjective insomnia. It comprises eight items and each item is rated on a scale of 0 to 3, with 0 indicating no problem and 3 indicating a very serious problem. Participants were instructed to score each item positively if they had experienced the specified sleep difficulty ≥ three times per week in the past month. The total AIS score ranges between 0 and 24, with a score ≥ 6 indicating the presence of insomnia [21]. With a Cronbach’s α of 0.81, the AIS has been reported to have good internal consistency [20].

#### SF-36

The SF-36, which comprises eight subscales [physical functioning, role limitations due to physical health problems, bodily pain, general health, vitality (energy/fatigue), social functioning, role limitations due to emotional problems, and mental health], was used to assess QoL. The physical dimensions (the first four subscales) make up the Physical Component Summary (PCS), whereas the Mental Component Summary (MCS) is derived from the remaining dimensions [22]. Both the PCS and MCS scores are continuous variables ranging from 0 to 100, with higher scores indicating a better health status. Specifically, very high PCS scores indicate no physical limitations, barriers, or reductions in well-being, and high energy levels, whereas very low scores indicate significant limitations (such as severe body pain or frequent fatigue) hindering self-care, physical, social, and role activities. Similarly, very high MCS scores indicate frequent positive emotions and a lack of psychological distress and limitations in ordinary social/role activities caused by emotional problems, whereas very low scores indicate significant social and role impairment due to frequent psychological distress and emotional issues [23]. The Chinese version of the SF-36 scale showed good internal consistency, with Cronbach’s alphas of 0.92, 0.90, and 0.85 for the overall scale, PCS subscale, and MCS subscale, respectively [24].

### Data analysis

All statistical analyses were performed using SPSS 25.0 (IBM Corp) and GraphPad Prism was used to generate graphs. All baseline characteristics were reported through descriptive statistics. Continuous variables were presented as Mean ± Standard Deviation (SD), while non-continuous variables were expressed as medians and Interquartile Ranges (IQL; P_25_ and P_75_). The Shapiro-Wilk test was used to assess normality, and comparisons between two groups were conducted using independent sample t-tests, while paired sample t-tests were used for within-group comparisons when the data were normally distributed. The rank sum test was employed in cases of non-normally distributed data. The chi-square or Fisher’s test was used to analyze the count data. Results or differences with *P* < 0.05 were considered statistically significant.

## Results

### Demographic and clinical characteristics

Table 1 shows patient distribution based on personal and clinical characteristics. Regarding general demographic information, patients in the Internal Fixation Group (IFG) were 33 males and 24 females with an average age of (37.77 ± 8.82) years and an average BMI of (22.27 ± 2.33) kg/m^2^. Forty-one (71.9%) were married, the majority resided in urban areas (73.7%), and 68.4% attained a high school education or higher. On the other hand, patients in the External Fixation Group (EFG) were 23 males and 16 females with an average age of (40.13 ± 9.08) years and a mean BMI of (22.97 ± 2.77) kg/m^2^. Twenty-one (53.8%) were married, most of them lived in urban areas (74.4%), and 66.7% attained a high school education or higher. Clinically, falls were the primary cause of fracture injury in the IFG (54.4%) and EFG (61.6%). Furthermore, compared to the IFG, operative time (69.30 (65, 75) vs. 39.62 (35, 45)), incision length (5.47 (5.0, 6.0) vs. 0.96 (0.5, 1.0)), and operative blood loss (35.70 (30.0, 42.5) vs. 21.28 (15.0, 30.0)) were significantly lower in the EFG (*P* < 0.001). Conversely, no statistically significant differences in AO classification, side of hand, time interval from injury to surgery, and length of hospital stay were found between the two groups (*P* > 0.05).

**Table 1:** Distribution of patients by personal and clinical characteristics

Characteristics categories	IFG (n = 57)	EFG (n = 39)	P
Age (year) (Mean ± SD)	37.77 ± 8.82	40.13 ± 9.08	0.207
BMI (kg/m^2^) (Mean ± SD)	22.27 ± 2.33	22.97 ± 2.77	0.184
Gender n (%)			
Male	33 (57.9%)	23 (59.0%)	
Female	24 (42.1%)	16 (41.0%)	0.916
Family location n (%)			
Rural	15 (26.3%)	10 (25.6%)	
Urban	42 (73.7%)	29 (74.4%)	0.941
Education level n (%)			
Less than high school education	18 (31.6%)	13 (33.3%)	
High school education and above	39 (68.4%)	26 (66.7%)	0.857
Marital status n (%)			
Unmarried	9 (15.8%)	9 (23.1%)	
Married	41 (71.9%)	21 (53.8%)	
Divorced	5 (8.8%)	7 (18.0%)	
Widowed	2 (3.5%)	2 (5.1%)	0.293
AO classification n (%)			
B2	10 (17.5%)	7 (18.0%)	
B3	12 (21.1%)	8 (20.5%)	
C1	17 (29.8%)	14 (35.9%)	
C2	18 (31.6%)	10 (25.6%)	0.908
Cause of injury n (%)			
Machine rolling injury	10 (17.5%)	5 (12.8%)	
Car accident injury	16 (28.1%)	10 (25.6%)	
Fall injury	31 (54.4%)	24 (61.6%)	0.745
Side of hand n (%)			
Left	26 (45.6%)	18 (46.2%)	
Right	31 (54.4%)	21 (53.8%)	0.958
Interval from injury to surgery (days) (Mean ± SD)	3.96 ± 0.91	3.92 ± 0.84	0.819
Operative time (min) Mean (P_25_, P_75_)	69.30 (65,75)	39.62 (35,45)	< 0.001
Incision length (cm) Mean (P_25_, P_75_)	5.47 (5.0,6.0)	0.96 (0.5,1.0)	< 0.001
Operative blood loss (ml) Mean (P_25_, P_75_)	35.70 (30.0,42.5)	21.28 (15.0,30.0)	< 0.001
Length of hospital stay (days) Mean (P_25_, P_75_)	5.86 (5.0,6.5)	5.51 (5.0,6.0)	0.583

*Notes* IFG: Internal Fixation Group; EFG: External Fixation Group; BMI: Body Mass Index

### Patients’ pain levels

**Fig. 1:**
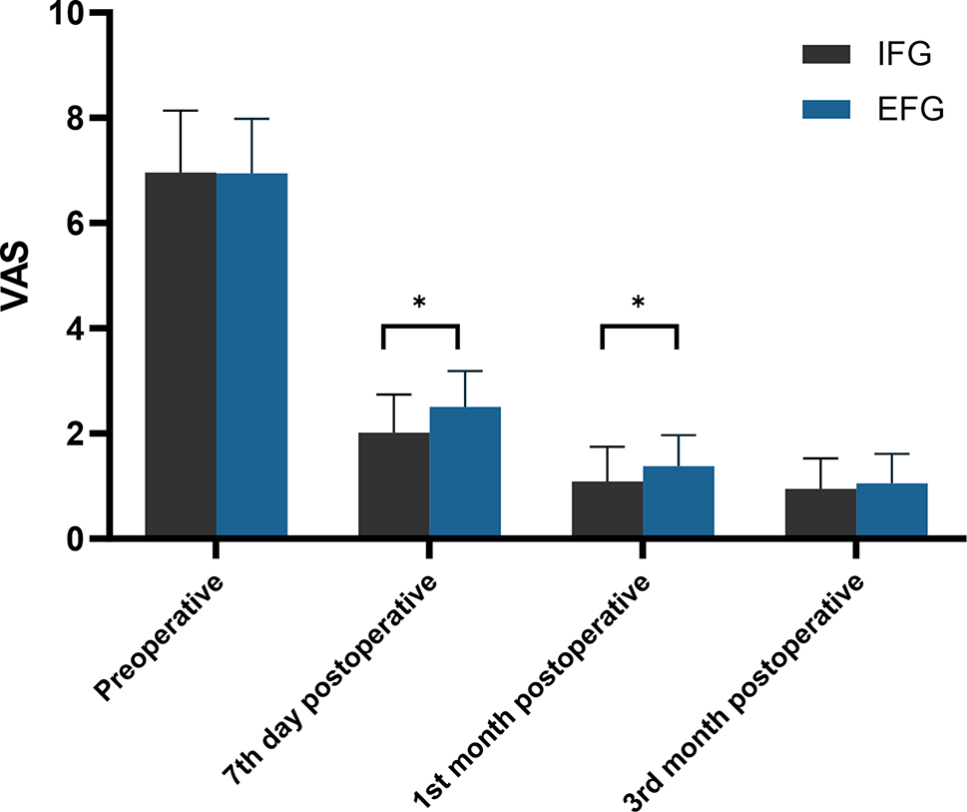
Distribution of mean and standard deviation of VAS for both groups over time. *Notes* VAS: The Visual Analog Scale; IFG: Internal Fixation Group; EFG: External Fixation Group; *: *P* < 0.05

We used the VAS pain scores to evaluate somatic pain in both patient groups (Table 2; Fig. 1). Our analysis revealed no statistically significant differences in VAS scores between the preoperative assessments (6.96 ± 1.18 vs. 6.95 ± 1.03, *P* = 0.945), and those in the third month postoperatively (0.95 ± 0.58 vs. 1.05 ± 0.56, *P* = 0.384). However, compared to the EFG, the IFG had significantly lower VAS scores on day seven post-surgery (2.02 ± 0.72 vs. 2.51 ± 0.68, *P* = 0.001) and the first month postoperatively (1.09 ± 0.66 vs. 1.38 ± 0.59, *P* = 0.027). Notably, both groups’ VAS scores decreased significantly over time, from the preoperative period to the third month postoperatively (*P* < 0.05).

**Table 2:** Measurements of pain for patients

	IFG (n = 57)	EFG (n = 39)	t	P
Preoperative	6.96 ± 1.18	6.95 ± 1.03	0.07	0.945
7th day postoperative	2.02 ± 0.72	2.51 ± 0.68	-3.38	0.001
1st month postoperative	1.09 ± 0.66	1.38 ± 0.59	-2.25	0.027
3rd month postoperative	0.95 ± 0.58	1.05 ± 0.56	-0.87	0.384

*Notes* IFG: Internal Fixation Group; EFG: External Fixation Group

### Patients’ anxiety levels

**Fig. 2:**
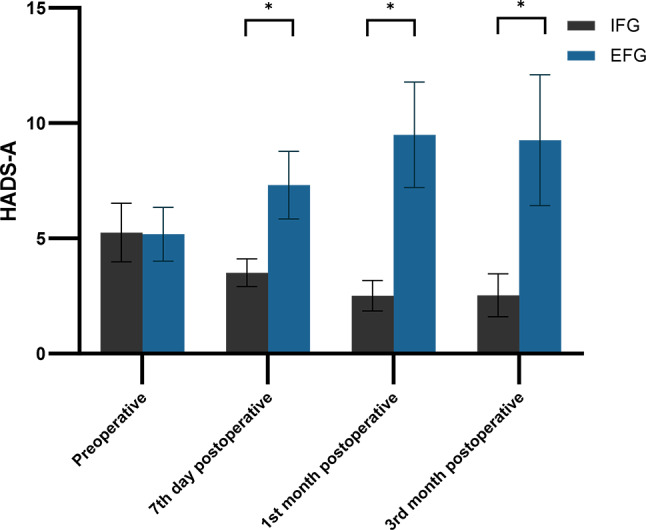
Distribution of mean and standard deviation of HADS-A for both groups over time. *Notes* HADS-A: Hospital Anxiety and Depression Scale-Anxiety; IFG: Internal Fixation Group; EFG: External Fixation Group; *: *P* < 0.05

Both groups exhibited no significant anxiety before surgery (5.25 ± 1.27 vs. 5.18 ± 1.17, *P* = 0.796). However, compared to the IFG patients, the EFG patients demonstrated significantly higher anxiety levels at day seven (3.51 ± 0.60 vs. 7.31 ± 1.47), first month (2.51 ± 0.66 vs. 9.49 ± 2.29), and third month (2.53 ± 0.93 vs. 9.26 ± 2.84) postoperatively, (*P* < 0.001). Notably, the EFG patients had the highest anxiety scores at the first month post-surgery (Table 3). Furthermore, there was a notable increase in the mean Hospital Anxiety and Depression Scale-Anxiety (HADS-A) scores in the EFG from the preoperative period to the first month postoperatively, whereas those in the IFG exhibited a significant decrease (*P* < 0.05). No statistically significant difference was found in the mean HADS-A scores between the two groups from the first to the third month postoperatively (*P* > 0.05) (Fig. 2).

### Patients’ depression levels

**Fig. 3:**
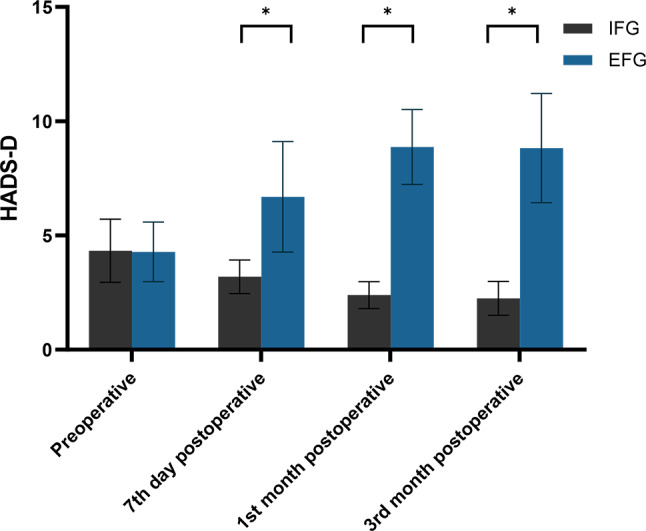
Distribution of mean and standard deviation of HADS-D for both groups over time. *Notes* HADS-D: Hospital Anxiety and Depression Scale-Depression; IFG: Internal Fixation Group; EFG, External Fixation Group; *: *P* < 0.05

No statistically significant difference was observed between the two groups in the mean Hospital Anxiety and Depression Scale-Depression (HADS-D) scores when assessing the patients’ depression severity during the preoperative period (4.28 ± 1.30 vs. 4.33 ± 1.38, *P* = 0.855). However, the mean HADS-D scores of the EFG patients were notably higher than those of the IFG patients at day seven post-surgery (6.69 ± 2.42 vs. 3.19 ± 0.74, *P* < 0.001), as well as first (8.87 ± 1.64 vs. 2.39 ± 0.59, *P* < 0.001 and third months postoperatively (8.82 ± 2.39 vs. 2.25 ± 0.74, *P* < 0.001) (Table 3), with the highest HADS-D scores observed in the EFG at the first month after surgery (Fig. 3).

### Patients’ sleep levels

**Fig. 4:**
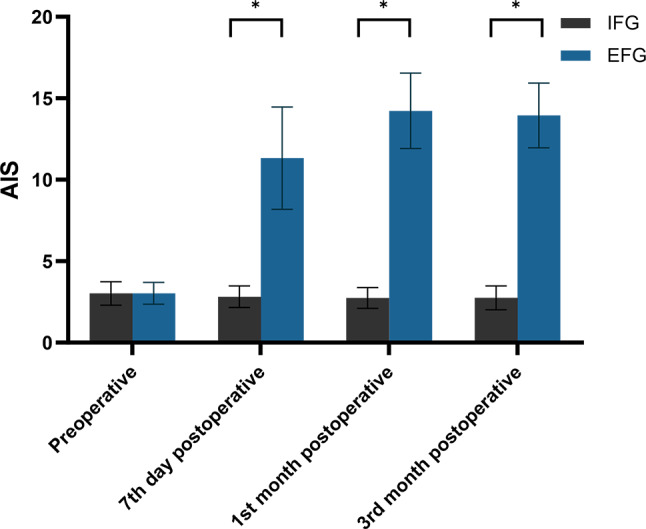
Distribution of mean and standard deviation of AIS for both groups over time. *Notes* AIS: Athens Insomnia Scale; IFG: Internal Fixation Group; EFG, External Fixation Group; *: *P* < 0.05

No statistically significant difference was found in the total AIS scores between the two groups before surgery (3.03 ± 0.67 vs. 3.02 ± 0.72, *P* = 0.956). However, compared to the IFG, the total AIS scores in the EFG were markedly higher at day seven (11.33 ± 3.14 vs. 2.82 ± 0.66, *P* < 0.001), and the first (14.23 ± 2.31 vs. 2.74 ± 0.64, *P* < 0.001) and third (13.95 ± 1.99 vs. 2.75 ± 0.74, *P* < 0.001) months post-surgery, with these differences being statistically significant. Furthermore, the EFG had the most significant increase in total AIS scores at day seven postoperatively and the highest total AIS score at the first month post-surgery (Table 3; Fig. 4).

### Patients’ PCS levels

**Fig. 5:**
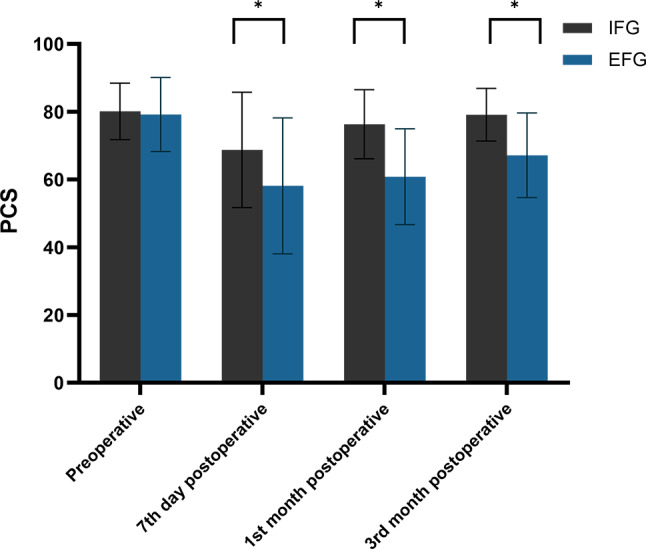
Distribution of mean and standard deviation of PCS for both groups over time. *Notes* PCS: Physical Component Summary; IFG: Internal Fixation Group; EFG, External Fixation Group; *: *P* < 0.05

We examined the average PCS scores of the two patient groups and found that although the mean PCS scores of both groups were higher in the preoperative period, there was no significant difference between them (80.11 ± 8.34 vs. 79.17 ± 10.92, *P* = 0.633). However, compared to the EFG patients, the mean PCS score was notably higher in the IFG patients at day seven (68.73 ± 17.00 vs. 58.11 ± 20.05, *P* = 0.006), and the first (76.29 ± 10.18 vs. 60.83 ± 14.16, *P* < 0.001), and third (79.12 ± 7.77 vs. 67.15 ± 12.50, *P* < 0.001) months postoperatively (Table 3; Fig. 5). Evidently, there was a positive change in the mean PCS score in both patient groups post-surgery.

### Patients’ MCS levels

**Fig. 6:**
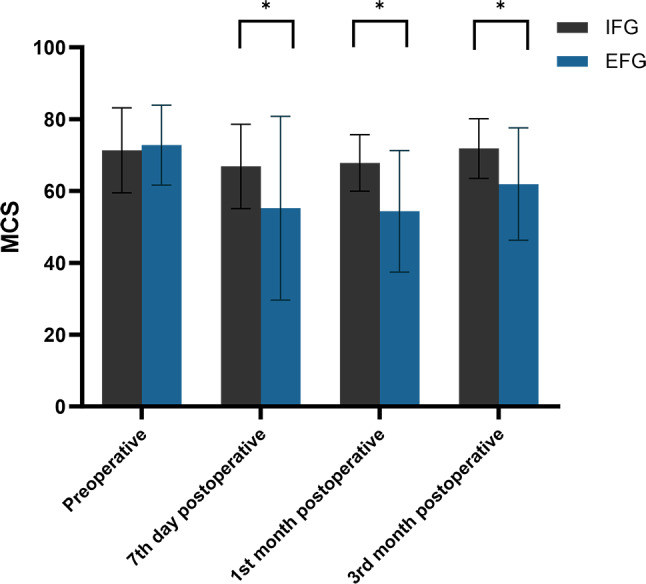
Distribution of mean and standard deviation of MCS for both groups over time. *Notes* MCS: Mental Component Summary; IFG: Internal Fixation Group; EFG, External Fixation Group; *: *P* < 0.05

Consistent with the mean PCS score results, no statistically significant difference was found in the mean MCS scores of the two patient groups in the preoperative period (71.33 ± 11.85 vs. 72.79 ± 11.13, *P* = 0.547). However, the mean MCS scores of the IFG were significantly higher than those of the EFG at the seventh day (66.85 ± 11.76 vs. 55.23 ± 25.58, *P* = 0.011) and the first (67.82 ± 7.88 vs. 54.35 ± 16.92, *P* < 0.001) and third months postoperatively (71.84 ± 8.32 vs. 61.93 ± 15.64, *P* = 0.001). Additionally, although the mean MCS score of the EFG patients was the lowest at the first month post-surgery, we observed a positive change in the overall trend of MCS scores in both patient groups throughout the postoperative period (Table 3; Fig. 6).

**Table 3:** Measurements of anxiety, depression, sleep, mental and physical component for patients

	IFG (n = 57)	EFG (n = 39)	t	P
HADS-A				
Preoperative	5.25 ± 1.27	5.18 ± 1.17	0.26	0.796
7th day postoperative	3.51 ± 0.60	7.31 ± 1.47	-15.27	< 0.001
1st month postoperative	2.51 ± 0.66	9.49 ± 2.29	-18.49	< 0.001
3rd months postoperative	2.53 ± 0.93	9.26 ± 2.84	-14.31	< 0.001
HADS-D				
Preoperative	4.33 ± 1.38	4.28 ± 1.30	0.18	0.855
7th day postoperative	3.19 ± 0.74	6.69 ± 2.42	-8.76	< 0.001
1st month postoperative	2.39 ± 0.59	8.87 ± 1.64	-23.66	< 0.001
3rd months postoperative	2.25 ± 0.74	8.82 ± 2.39	-16.62	< 0.001
AIS				
Preoperative	3.02 ± 0.72	3.03 ± 0.67	-0.06	0.956
7th day postoperative	2.82 ± 0.66	11.33 ± 3.14	-16.67	< 0.001
1st month postoperative	2.74 ± 0.64	14.23 ± 2.31	-30.27	< 0.001
3rd months postoperative	2.75 ± 0.74	13.95 ± 1.99	-33.64	< 0.001
PCS				
Preoperative	80.11 ± 8.34	79.17 ± 10.92	0.48	0.633
7th day postoperative	68.73 ± 17.00	58.11 ± 20.05	2.79	0.006
1st month postoperative	76.29 ± 10.18	60.83 ± 14.16	5.86	< 0.001
3rd months postoperative	79.12 ± 7.77	67.15 ± 12.50	5.32	< 0.001
MCS				
Preoperative	71.33 ± 11.85	72.79 ± 11.13	-0.60	0.547
7th day postoperative	66.85 ± 11.76	55.23 ± 25.58	2.65	0.011
1st month postoperative	67.82 ± 7.88	54.35 ± 16.92	4.64	< 0.001
3rd months postoperative	71.84 ± 8.32	61.93 ± 15.64	3.62	0.001

*Notes* IFG: Internal Fixation Group; EFG: External Fixation Group; HADS-A: Hospital Anxiety and Depression Scale-Anxiety; HADS-D: Hospital Anxiety and Depression Scale-Depression; AIS: Athens Insomnia Scale; PCS: Physical Component Summary; MCS: Mental Component Summary

## Discussion

Most orthopedic surgeons are currently focused on the physical aspect of healing, which is the primary concern for fracture patients [26–28]. On the other hand, the psychological dimension is frequently overlooked in the realm of fracture surgery. This is particularly true regarding surgical approach selection, which often entails choosing between internal fixation and external fixation operations. Herein, we clinically found that in DRF patients, the EFG had shorter operation times, smaller incision lengths, and less intraoperative blood loss than the IFG. However, regarding VAS pain scores, the IFG showed more significant pain reduction than the EFG at seven days and one-month post-surgery. There was no difference between the two methods in healing rates [26]. Conversely, significant variations were found in the patients’ long-term psychological states postoperatively, potentially due to the differing surgical approaches. Specifically, compared to the IFG patients, the EFG patients were more prone to negative emotions such as anxiety and depression, as well as insomnia at seven days, one month, and three months postoperatively. Additionally, while there were significant differences in PCS and MCS scores between the two groups at seven days, one month, and three months postoperatively, both groups showed positive change over time.

Internal and external fixation are both excellent therapeutic modalities, especially after decades of development in fracture treatment. Each has its advantages based on different indications. External fixation is crucial for open fractures, given its simplicity, flexibility, and minimal tissue damage. It has been widely used in clinical practice and has particularly demonstrated benefits in treating limb fractures with poor soft tissue conditions [29]. Moreover, external fixation significantly reduces complications such as infections as well as skin and soft tissue necrosis that could result from open reduction internal fixation, making it indispensable in limb lengthening procedures and the treatment of limb fractures with severe soft tissue injury and infection [30]. The intersection of indications for both methods has gradually expanded with continuous advancements in orthopedic medical concepts (external and internal fixations), especially with the introduction of the “Biological Osteosynthesis (BO)” principle [31, 32], leading to more controversies related to surgical method selection. However, when treating DRF, we believe that the choice of fixation method should still be based on the specific circumstances of the fracture. When there is significant shortening of the radius and partial metaphyseal bone loss or comminution, or in cases of compressive fractures, external fixation with appropriate traction to maintain or restore the height of the radius can be employed. This approach is particularly suitable for middle-aged and elderly patients, as it can avoid the need for a second surgery that might be required with internal fixation. Conversely, internal fixation is generally preferred when there is no significant shortening of the radius. Previous research [33–35] has often focused on exploring the physiological treatment effects while overlooking the psychological aspects. Offering a new angle to elucidate the respective impacts on patient emotions postoperatively, this study evaluates the psychological status of patients who have undergone internal and external fixation over multiple periods. Besides offering orthopedic surgeons a secondary basis for surgical method selection beyond absolute indications, this analysis provides a reference for the optimal timing of psychological interventions when patients experience adverse emotions.

According to previous research [36–39], external fixation devices can effectively control the position and stability of fractures, thereby promoting healing. However, these external fixators must often be maintained on the skin for some time, which may cause discomfort and pain, as well as a high probability of pin tract infections [40, 41], consequently leading to psychological discomfort for the patients. Furthermore, patients may feel unattractive due to external fixators, affecting their self-esteem, sleep, and psychological state [13, 42, 43]. Our results also indicate that the EFG patients are more likely to experience negative emotional reactions such as anxiety, worry, irritability, depression, and feelings of inferiority in the first three months post-surgery. Follow-up with a large patient cohort revealed that these negative emotional reactions are mainly associated with various factors related to the inconvenience and discomfort of wearing the external fixator. Additionally, practical concerns such as restricted mobility, difficulty bathing and dressing, and judgmental looks from others can further exacerbate the negative emotions in trauma patients, with severe cases even leading to suicidal thoughts [14, 15]. Moreover, patients’ sleep quality (as manifested by easy awakenings at night, poor nighttime sleep quality, and shortened total sleep time) could be affected [44]. During the interviews, we observed more pronounced anxiety, depression, and insomnia in EFG patients who were the primary source of family income and experienced financial difficulties. This area of concern will require additional research in the future. On the other hand, patients who underwent internal fixation surgery did not show significant anxiety, depression, or insomnia. This finding may be because internal fixation patients experienced less pain and discomfort, resulting in less significant negative psychological impacts compared to external fixation patients [45]. Additionally, patient acceptance is an important aspect. Engaging in manageable activities during postoperative recovery is crucial for the patients’ psychological adjustment. Internal fixation patients can participate in a more diverse range of social activities post-surgery, to some extent mitigating the impact of the operation on their QoL and alleviating anxiety and depression [45, 46].

A period of emotional accumulation is often required for psychological changes. From a temporal perspective, patients perceive the most significant increase in indicators such as HADS and AIS to occur at seven days postoperatively compared to preoperatively. Although patients are comprehensively informed about the surgical procedure beforehand, witnessing the Schanz screws directly connected to the body surface is initially difficult to accept. Therefore, some patients may experience stress and anxiety, and most patients may postoperatively experience sleep disturbances due to the subconscious protection of the external fixation frame. Sleep disturbances can set off a vicious cycle of low mood and worrying about the illness.

Patients exhibit the most prominent signs of anxiety, depression, and insomnia at one month postoperatively. This indicates that clinical psychological interventions are most effective within the first month after fracture surgery. If circumstances allow, psychological counseling should be initiated immediately within the first week post-surgery when the HADS and AIS indicators rise the fastest. At this point, healthcare professionals could provide appropriate psychological support and counseling, enhancing the patients’ self-awareness and cognitive abilities, which in turn reduces their worries, fears, and feelings of depression, helping them to better adapt to the postoperative recovery process [47]. Moreover, Jacobs et al. found a significant correlation between psychological factors and the body’s recovery process [48]. In this regard, providing timely psychological treatment to patients not only holds social significance but also contributes to functional recovery. Certain external factors (including the prolonged wearing of external fixation devices and postoperative pain) gradually diminish in their impact on some patients after the first month postoperatively, leading to changes in the patients’ psychological state and sleep patterns. As a result, some patients could return to the hospital to get the external fixation devices loosened and may also gain confidence from their satisfactory recovery. The loosening of external fixation devices one month post-surgery involves adjusting the bilateral ball heads and the central connecting slide bar of the ORTHOFIX external frame. The aim is to facilitate early functional exercises, maintain the height of the radius, and prevent joint stiffness. Interestingly, the support provided by external fixation during these early exercises can lead to improved functional activity. For most patients with distal radial fractures, the external fixator is removed 1–2 months after surgery. However, there is a subset of patients whose fractures have not yet fully healed. These fractures, such as those at the distal radial metaphysis and the radial diaphyseal transition zone, tend to heal more slowly. For these patients, the time for removal of the fixator can be extended to three months. This extension is predicated on ensuring that patients carry out functional exercises. These patients require psychological counseling during follow-up visits. However, this psychological improvement may vary to some extent based on factors such as ethnicity, region, and age groups included herein.

Primarily based on patients’ subjective opinions, QoL measurement could be the most crucial approach for assessing their postoperative physical and psychological functional recovery [49]. Herein, we found that the IFG patients had higher PCS and MCS scores at seven days, one month, and three months postoperatively compared to the EFG patients. Specifically, the IFG patients had significantly higher scores in items related to moderate activities, shopping, bathing and dressing oneself, limitations in desired activities, and increased difficulty in completing tasks in the PCS section. They also had significantly higher scores in items related to reduced work hours due to emotional reasons, impact on normal interactions with family and friends due to emotional reasons, feeling down, and feeling exhausted in the MCS section. Furthermore, despite having the greatest increase in the PCS and MCS scores between the seventh day and the first month postoperatively, the EFG exhibited lower PCS and MCS scores at three months compared to preoperative levels. Other studies on postoperative QoL found that increases in PCS and MCS scores mostly occur within the first six months postoperatively [50, 51]. Based on stable reduction, both internal and external fixation could result in satisfactory fracture recovery in the short term postoperatively [4–8]. However, the time it takes for patients to return to their previous functional status in society varies. In addition to psychological factors, this discrepancy could be influenced by the practical impact of daily behavioral hindrances of wearing external fixation devices. Furthermore, patients wearing external fixation devices could subjectively feel they can still not engage in daily activities and perform regular duties.

According to previous research [52–54] patients with higher postoperative QoL tend to have higher happiness indices, are less susceptible to anxiety, depression, and insomnia, and are better positioned for physiological recovery, as well as better interpersonal and social interactions. External fixation can negatively impact patients’ postoperative QoL, leading to an increased incidence of low mood, fatigue, melancholy, and social limitations due to external scrutiny (such as reluctance to communicate with friends and family and reduced social activities). The duration of wearing an external fixator generally falls within an acceptable range for most DRF patients. However, compared to internal fixation, statistically significant negative emotional differences have been observed in external fixation. Special attention should be paid to monitoring emotional changes in patients requiring long-term external fixation procedures, such as bone lengthening [55]. An external fixator may lead to difficulties in daily activities, such as mobility issues and grooming. Therefore, healthcare professionals should offer practical assistance and guidance on dressing and other aspects of daily living to help patients better adapt to wearing an external fixator and adjust to their new life situation [56]. Moreover, guiding patients who have undergone external fixation surgery to actively participate in rehabilitation activities could help them better adapt to wearing the external fixator, boost their confidence and self-esteem, and reduce the incidence of negative emotions.

Our study primarily aimed to provide new insights into the selection of surgical procedures for fracture patients from multiple dimensions, including psychological status, sleep, and QoL. In conclusion, compared to external fixation surgery, internal fixation surgery has a smaller impact on the emotional state, sleep, and QoL of fracture patients during postoperative recovery. This outcome is mostly due to subjective acceptance of long-term wearing of external fixation devices, restricted daily activities, and longer recovery time. Notably, the above conclusion does not mean internal fixation is superior to external fixation. The two approaches have different indications, and external fixation has its irreplaceable advantages. It is crucial to recognize that the choice between external fixation and internal fixation often hinges on the specific characteristics and requirements of the fracture type, such as open versus closed fractures. This distinction in indications suggests a potential variability in the applicability of these treatments, which could influence the study’s outcomes. To address this, our analysis carefully considers the fracture types and conditions across patient groups to minimize potential biases. However, orthopedic surgeons must seriously consider the potential psychological impact of external fixation on patients’ post-surgery. The results of a randomized controlled trial led by the Major Extremity Trauma Research Consortium (METRC) suggest that even in the case of severe open tibial fractures, the routine use of modern external ring fixation for treatment should be avoided. Patients with poor psychological baselines may require timely psychological intervention if negative emotions worsen within the first week following external fixation treatment [57]. Notably, internal fixation surgery may be a better choice when both internal and external fixation can be performed on a patient with a fragile psychological health status. In such cases, surgical outcomes and risks, as well as individual patient factors, should remain the primary considerations. Overall, our study findings provide insight into the psychological impact of different fixation procedures on fracture patients. Doctors should be aware of the potential external fixation-induced adverse psychological events and provide patients with timely intervention and the best treatment plan possible.

This study has certain limitations. First, the sample size was relatively small, necessitating multi-center studies with larger sample sizes to better generalize the findings in the future. Second, recall bias was inevitable despite using well-validated questionnaires such as HADS and SF-36. Third, the overall patient compliance decreased after the removal of the implant despite our efforts to conduct long-term patient follow-ups at six months postoperatively and beyond, making it difficult to assess the patients’ psychological status over a longer period. Based on these drawbacks, future research could explore the impact of internal fixation and external fixation surgeries on the psychological status of patients with various types of fractures, as well as different age and gender groups, and consider longer follow-up periods.

## Conclusion

This study found that in the early postoperative period, internal fixation surgery did not have a significant effect on negative emotions such as anxiety and depression compared to external fixation surgery, and patients showed better physical and psychological recovery during the postoperative rehabilitation period. In cases without absolute indications, the selection of a surgical approach should factor in its impact on patients’ psychological well-being as part of the treatment plan. Furthermore, individuals who undergo external fixation treatment and exhibit significant anxiety and depression should receive prompt psychological counseling in the early stages to mitigate the risk of developing severe anxiety and depression disorders.

## Data Availability

The datasets used and/or analyzed during the current study are available from the corresponding author on reasonable request.

## Abbreviations

DRF: Distal Radius Fractures
QoL: Quality of Life
VAS: Visual Analog Scale
HADS: Hospital Anxiety and Depression Scale
AIS: Athens Insomnia Scale
SF-36: Medical Outcomes Study Short Form 36
IFG: Internal Fixation Group
EFG: External Fixation Group
PCS: Physical Component Summary
MCS: Mental Component Summary
BMI: Body Mass Index
SD: Standard Deviation
IQL: Interquartile Ranges
HADS-A: Hospital Anxiety and Depression Scale-Anxiety
HADS-D: Hospital Anxiety and Depression Scale-Depression
BO: Biological Osteosynthesis
METRC: Major Extremity Trauma Research Consortium
